# New species and records of ortholasmatine harvestmen from México, Honduras, and the western United States (Opiliones, Nemastomatidae, Ortholasmatinae)

**DOI:** 10.3897/zookeys.52.471

**Published:** 2010-07-30

**Authors:** William A. Shear

**Affiliations:** Department of Biology, Hampden-Sydney College, Hampden-Sydney VA 23943 USA

**Keywords:** Ortholasma, Dendrolasma, Trilasma, Cladolasma, Halitherses, amber, fossil, California, Sierra Nevada, Nuevo León, Tamaulipas, Hidalgo, Veracruz, Honduras, Japan, Thailand, new species, new combination

## Abstract

The genus Trilasma Goodnight & Goodnight, 1942 is reinstated for Mexican ortholasmatines, and Cladolasma Suzuki, 1963 is reinstated for two species from Japan and Thailand, Cladolasma parvula Suzuki, **comb. n.** and Cladolasma angka (Schwendinger & Gruber), **comb. n.** Eight new species in the subfamily Ortholasmatinae Shear & Gruber, 1983 are described, as follows: Ortholasma colossus **sp. n.** is from California, Trilasma tempestado **sp. n.**, Trilasma hidalgo **sp. n.**, Trilasma trispinosum **sp. n.**, Trilasma ranchonuevo **sp. n.**, Trilasma petersprousei **sp. n.** and Trilasma chipinquensis, **sp. n.** are from México, and Trilasma tropicum **sp. n.** from Honduras, the farthest south for a dyspnoan harvestman in the New World. A new distribution record for Martensolasma jocheni Shear 2006 is given. The recently described Upper Cretaceous amber fossil Halitherses grimaldii Giribet & Dunlop 2005 is not a member of the Ortholasmatinae, but is likely a troguloidean of an undiagnosed family.

## Introduction

The harvestman subfamily Ortholasmatinae was monographed by Shear and Gruber (1983). That work focused perforce on the western United States, whence came the majority of material available for study, and the seeming center of diversity for the two recognized genera of the subfamily, Ortholasma Banks, 1894 and Dendrolasma, Banks 1894. Outside the US, a single species of Dendrolasma was known at that time from Japan, and two Ortholasma species had been recorded from México.

Since 1983, there has been little activity in the study of these most unusual harvestmen, but a few years after the 1983 monograph’s publication, Shear and Gruber (1987) found that Ortholasma setulipes Shear & Gruber, 1987, from southern California, was a synonym of Ortholasma coronadense Cockerell, 1916. Schwendinger and Gruber (1992) later described a fourth species of Dendrolasma from Thailand. More recently, Giribet and Dunlop (2005) postulated a fossil record for ortholasmatines going back to the Upper Cretaceous (Albian; ca. 100 million years ago), but their fossil species from Burmese amber, Halitherses grimaldii Giribet & Dunlop, 2005, lacks both the characteristic tergal ornamentation and eye-tubercle projection, and has no clear synapomorphies of Ortholasmatinae. It is clearly a troguloid of some kind, but not likely a member of any extant family. Finally, Shear (2006) reported on a new genus and species of ortholasmatine (Martensolasma jocheni Shear, 2006) from Aguascalientes, México.

Ortholasmatines have received some attention in studies of harvestman phylogeny. Giribet et al. (2002) presented molecular evidence for a relationship between Nemastomatinae Simon, 1872 and Ortholasmatinae, but a more recent study (Giribet et al. 2010), including more loci and denser taxonomic sampling, analyzed under direct parsimony optimization, showed Nemastomatidae as paraphyletic, and ortholasmatines sister to a clade including Trogulidae Sundevall, 1833, Dicranolasmatidae Simon, 1879 and Nipponopsalididae Martens, 1976. On the other hand, the same data analyzed by maximum likelihood showed a monophyletic Nemastomatidae, including the ortholasmatines. Giribet et al. (2009) call for further sampling and analysis in the Dyspnoi, and it would be premature to make any taxonomic changes based on these studies.

Shear and Gruber (1983) took a conservative approach in grouping the species of ortholasmatines into only two genera, Dendrolasma and Ortholasma. Suzuki, (1963) had originally described the species Dendrolasma parvulum in a different genus, Cladolasma Suzuki 1963, only later synonymizing this genus with Dendrolasma (Suzuki 1974). Schwendinger and Gruber, (1992) argued that because of distinctive characters their new species from Thailand (Dendrolasma angka Schwendinger & Gruber 1992) shared with Dendrolasma parvulum, the genus Cladolasma might well be revalidated. However, they did not take that formal step because males had not been collected (and are still unknown). After restudying the matter based on new specimens and some of the same material available in 1983, I agree with Schwendinger and Gruber (1992), and in this paper revalidate the generic name Cladolasma Suzuki, 1963, for Cladolasma parvulum and Cladolasma angka. Like Schwendinger and Gruber (1992), I fully anticipate a wealth of new species in this genus to arise from ongoing exploration of the montane forests of southern China, Laos, Vietnam and Thailand.

The first Mexican species of ortholasmatine, previously Ortholasma (Trilasma) bolivari (Goodnight & Goodnight 1942), was placed by Goodnight and Goodnight, (1942) in a new genus, Trilasma Goodnight & Goodnight 1942, which Shear and Gruber (1983) retained as a subgenus under Ortholasma. The appearance of several new species from México, all sharing the diagnostic characters of subgenus Trilasma, leads me to restore that taxon to full generic level for all the Mexican species, and a new one from Honduras.

I also describe in this paper a new Californian species from the Sierra Nevada, including Sequoia National Park. Ortholasma colossus, new species, occurs at the southern end of the Sierra Nevada distribution of Ortholasma levipes Shear & Gruber 1983, and a single specimen of the new species was mentioned and briefly characterized in Shear and Gruber’s monograph. The collection of much additional material makes possible the description of this, the largest of all ortholasmatines, nearly half again as long as the previously known largest species.

Though Shear and Gruber, (1983) had available numerous samples of most of the species they studied (except, for example, the troglobiont Trilasma sbordonii (Šilhavý 1974), still known only from a few specimens), most of the new species below are described from single specimens or a small number of specimens. However, the earlier monographic study gave a very clear picture of the ranges of variation to be expected in species of the subfamily, and in each of the cases below, I am confident that the new taxa lie outside those ranges as they are understood in better known congeners. In particular, the new Mexican species are mostly geographically distant from the distribution of Trilasma bolivari and Trilasma sbordonii, the previously known Mexican species, and are easily distinguished from them and from each other. The new Honduran species, Trilasma tropicum, is distinctive and represents an extension of the subfamily into the New World humid tropics, just as Cladolasma angka pushed the range southward in Asia. With the discovery of this species, ortholasmatines are now known from very near the border between Alaska and British Columbia south to Honduras.

Shear and Gruber (1983) presented an admittedly confusing scenario for the historical biogeography of the ortholasmatines. However, they were clear about proposing an origin for the subfamily in the central Mexican highlands, or Transverse Volcanic Belt, with subsequent dispersal northward into northwestern North America and ultimately far eastern Asia. That hypothesis is strengthened by the documentation in this study (and in Shear 2006) of considerable additional diversity in México, close to the supposed region of origin, which includes Martensolasma jocheni Shear, 2006, a species lacking the unusual modifications of the eye tubercle found in all other species, as well as exhibiting a simple pattern of cuticular sculpture and a penis from which it would be possible to derive the more apomorphic forms found in all other ortholasmatines. The new species far to the south in Honduras suggests that during appropriate climatic regimes, perhaps glacial maxima, ortholasmatines also spread southward; this is reinforced by the fact that Trilasma tropicum sp. n. is characterized by a narrow median hood process inserted dorsally on the eye tubercle, rather than a broad hood process arising from the rostral side of the eye tubercle.

Ortholasmatines are the most southerly occurring members of the harvestman suborder Dyspnoi, which appears to be limited to the northern hemisphere. In many cases these harvestmen are clearly associated with boreal regions or relatively high elevations, and indeed such is the case with ortholasmatines, though they seem tolerant of drier habitats so long as temperatures are moderate to low. With the exception of Ortholasma pictipes Banks, 1911, and the species of Dendrolasma, which are found in moist coniferous forests from northern California to Alaska, North American Ortholasma species are often characteristic of sclerophyll vegetation (though collecting notes suggest they are found even there in moister microhabitats, such as caves and canyons). There may be additional species awaiting discovery in such habitats in southern California, and the diversity of the subfamily in México may be significantly greater than presently documented. Found near sea level in northwestern North America, this proclivity for cool temperature regimes carries ortholasmatines to high elevations in México, where Trilasma species are found in coniferous forests mostly above 2100 m (7000’) elevation, or in cloud forests. Even at these elevations, collection data suggest that some tropical and subtropical species are troglophiles, seeking out the damper, cooler conditions in caves. In the Sierra Nevada of California, individuals are often taken in caves, but show no troglomorphic adaptations, and while two of the new species described below from México are from caves and are conspicuously long-legged, they do not appear to be troglobionts. The caves have been much more fully explored than their surface environs, so the number of records of ortholasmatines from caves in California and México is probably more an artifact of collecting than an indicator of habits.

### Is the fossil Halitherses grimaldii an ortholasmatine?

Giribet and Dunlop (2005) published a detailed description of a remarkably well-preserved fossil harvestman species from 100 myr-old Burmese (Myanmar) amber. Their new genus and species, Halitherses grimaldii, was based on two specimens found in the same small block of amber. It was not possible to determine the sex of the specimens. They tentatively assigned the taxon to Ortholasmatinae. The presence of clavate glandular setae (“Kugelhaare” of Wachmann 1970; see below for a note on these setae) on the clawless palpi places the species in the superfamily Troguloidea, and its general appearance suggests Nemastomatidae, except for the extraordinarily large eye tubercle. However, the specimens show no synapomorphies of the subfamily Ortholasmatinae, and the arguments for placing the species there are based on misunderstandings. Giribet and Dunlop (2005) refer to “the forward-projecting eye tubercle, the heavy cuticular ornamentation, and the fused meso- and metapeltidia….”. However, the eye tubercle of ortholasmatines does not project forward at all but is simple and moundlike. The anterior projection (median hood process) is an extension from the dorsal or rostral  surface; no such projection is seen in Halitherses grimaldii, which has a very large caddid-like eye tubercle with a median depression, that resembles no known nemastomatid. Unlike the small eyes of nemastomatids, the eyes of Halitherses grimaldii are inordinately large. The fossil, in short, exhibits no sign of the “troguloid facies” in which processes from the eye tubercle and forward margin of the carapace form a hood concealing the chelicerae and palpi, and in which the body is distinctly flattened. This facies is diagnostic of ortholasmatines.

The cuticular ornamentation of Halitherses grimaldii consists of low, rather rounded, densely scattered tubercles quite unlike the anvil-shaped, T-shaped, or multi-armed tubercles found in ortholasmatines, and indeed are more like the ornament of the ischyropsalidoid genus Hesperonemastoma. There is no organization of the ornamentation into keel cells, as seen in all ortholasmatines, even the single species lacking the median hood process. Likewise, lateral hood processes, universal in all ortholasmatines, are not seen in the fossil species. It is not clear what the authors mean by “fused meso- and metapeltidia” since it is obvious from their illustrations that at least the metapeltidium is free. The condition of the specimen and the angles of observation available make it difficult to see. No troguloideans have a free mesopeltidium, this “first thoracic segment” always being fused to the carapace, or propeltidium, and the free metapeltidium with scutum parvum is found in several troguloideans (Shultz and Pinto-da-Rocha 2007). It seems to me likely that Giribet and Dunlop are mistaken and that, as usual, the mesopeltidium is fused to the carapace (propeltidium) and the metapeltidium is free.

Giribet and Dunlop included Halitherses grimaldii in the morphological dataset of Giribetet al. (2002), and the result in a strict consensus cladogram was simply to group the species with all other included troguloids in a “comb” (fig. 6 of Giribet & Dunlop 2005). This was undoubtedly due to much missing data for the fossil species, but also showed no close relationship to Nemastomatidae.

All the evidence suggests to me that Halitherses grimaldii is not an ortholasmatine. The large eyes and eye tubercle are unique, and among troguloids, only the cavernicolous nemastomatids have such extraordinarly long legs. I think it highly likely that this species represents at the least a new, probably extinct, family within Troguloidea.

### Palpal setae

Two distinct types of palpal setae in the Dyspnoi, not necessarily homologous, have been confused in some recent literature, where both are referred to as “clavate” hairs or setae. In the superfamily Troguloidea, the palpal setae are of the type described in great detail by Rimsky-Korsakov (1924) and Wachmann (1970) as Kugelhaare, and to which I have previously referred as “Rimsky-Korsakov setae”. These setae are quite complex, broad at the base and set in special sockets; near the distal tip a radial array of small tubules emerges, not unlike the ribs of an umbrella, and from the openings at their tips, a sticky secretion is produced which is often seen as coagulated droplets on preserved specimens (Figs 6, 8, 43). Because these setae actually are club- or mace-shaped, they can correctly be called clavate (though Kugel [German] means “ball” [English]).

However, the palpal setae in some species of the superfamily Ischyropsalidoidea, illustrated by me in 1986 using scanning electron micrographs, are quite different. In these setae, fine tubules occupy at least the distal half of the seta, are not arranged radially, and may or may not be the source of a secretion. Similar setae are often found on the palpi of both juvenile and some adult Eupnoi (Shear 1986, 2004; Hunt and Cokendolpher 1991). This second type of seta might conveniently be termed “plumose,” as I did in 1986. Whatever name is finally adopted, it is clear from the electron micrography that the two types of seta are quite different, and may not be homologous as glandular setae.

Details need to be examined before these setae are used further as characters in phylogenetic reconstructions. For example, both types of hairs appear to be absent at least in adults of the ischyropsalidoid genera Ischyropsalis, Ceratolasma and Acuclavella. Crosbycus dasycnemus plumose setae appear different from those of Taracus or Sabacon; the former are truncate, not pointed, and the tubules appear not to extend all the way to the tip.

### Chemical defenses

Several specimens of Ortholasma pictipes and Ortholasma rugosum were dropped alive into small amounts of methanol, and the extract analyzed for volatile components. This method has proven itself as a way of determining the presence and nature of the chemical defenses of harvestmen (Jones et al. 2009; Shear, Jones et al. 2010; Shear, Snyder et al. 2010). In both species, no volatile compounds could be detected in the extract, suggesting strongly that at least two species of ortholasmatine lack the usual chemical defenses found in Opiliones. More results will be reported in detail in a later publication, but of five species, all in different genera, and three different families, of North American Dyspnoi studied, none had chemical defenses extractable by methanol, and at least one species may lack ozopores.

Recently, Raspotnig et al. (2010) have reported the first determination of the chemical defense of a dyspnoan, Paranemastoma quadripunctatum (Perty, 18330 (Nemastomatidae); they found an array of naphthoquinones and anthraquinones. At least the anthraquinones are usually solids at environmental temperatures, and Raspotnig et al. (2010) suggest they become available for defense only after being dissolved in enteric fluid regurgitated through the mouth. They found that in some dyspnoans, ozopores are present but cryptic, facing downward toward the mouth under the carapace, and this indeed may be the case in the species I examined—a second look is required. However, Raspotnig et al. (2010) were able to recover the same set of compounds using two methods, absorption on filter paper, and whole-body extraction. So our failure to find any secretions via whole-body extraction remains puzzling if in fact chemical defenses are present in the species we examined.

## Methods

Measurements of the appendages of single specimens were taken from material temporarily mounted in glycerine on microscope slides, using a calibrated ocular micrometer in an Olympus BX-50 compound microscope. Lengths and widths of appendage segments were taken from their greatest lengths and greatest widths; length of first cheliceromere includes the apodeme, that of the second cheliceromere the fixed finger. Total leg lengths do not include coxae or trochanters, and are the summed lengths of the femora, patellae, tibiae, metatarsi and tarsi. Body dimensions were measured under an Olympus SZH stereomicroscope, using an external scale and camera lucida attachment, and photomicrographs were taken using this microscope and a mounted Luminera Infinity 1 digital camera; photographs were manipulated for clarity in the programs PhotoShop and IPhoto. The animals in these photographs appear red or orange rather than their field color of brown or black because the use of transmitted light in taking the photographs enhanced the clarity of the keel cells, but incidentally produced the colors; removing the color results in less clarity.

Total length was measured from the distal tip of the median hood process to the posterior margin of the abdominal scute, excluding the extended keel tubercles; total width at the widest point of the body (usually near the posterior margin of the scute). Length of the median hood process was measured in dorsal view from the keel just posterior to the eye tubercle to the distal tip, and width was measured at the widest point. All measurements are in millimeters. Tarsal article counts vary, sometimes on the left and right sides of the same individual, but usually only by one or two articles. The counts given in the descriptions below should be taken as possibly varying by one or two in either direction.

Map coordinates and approximate elevations were obtained from Google Earth when the data did not appear on collection labels.

### Depositories:

TMMTexas Memorial Museum

AMNHAmerican Museum of Natural History

CASCalifornia Academy of Sciences.

## Taxonomy

Family Nemastomatidae Simon, 1872

### Subfamily Ortholasmatinae

Shear & Gruber, 1983

Ortholasmatinae Shear and Gruber 1983, p. 13 (see for pre-1983 references); Schwendinger & Gruber 1992, p. 60; Shear 2006, p. 192 (emended diagnosis).

#### Diagnosis.

Nemastomatid Opiliones with the anvil-shaped tubercles on the dorsum exaggerated, with long horizontal arms overlapping in patterns to form distinctive keel cells (Figs 2, 3); lateral anterior margins of the carapace with at least one (up to three) forward-projecting lateral hood process on either side of the eye tubercle; eye tubercle in all but one genus with long, forward-projecting median hood process bearing large T-shaped tubercles laterally and sometimes dorsally. All these characters are absent from nemastomatines.

#### Key to Genera

**Table:** 

1.	With a forward-projecting process on the eye tubercle, bearing elongate T-shaped tubercles laterally	2
–	Without such a process, eye tubercle with small, blunt dorsal spine; Aguascalientes, Puebla, México	Martensolasma jocheni Shear
2.	Frontal border of the carapace with a single long process on either side of the eye tubercle	3
–	Frontal border of the carapace with two or more such processes	4
3.	Metapeltidium separated from both carapace and abdominal scutum; keel cells small, not in transverse rows; southern Japan and SE Asia	Cladolasma Suzuki
–	Metapeltidium fused to abdominal scutum, partially fused to carapace; keel cells larger, arranged in approximate transverse rows; northern Pacific coast of North America	Dendrolasma Banks
4.	Dorsal hood process with median armed tubercles; scute with rows of small cells between rows of large ones; males without a gland on first cheliceromere; central and northwestern México to Honduras	Trilasma Goodnight & Goodnight
–	Dorsal hood process without median armed tubercles; only large cells on scute; males with dorsal gland on first cheliceromere; western North America from southern California to northern British Columbia	Ortholasma Banks

#### Martensolasma

Shear, 2006

Martensolasma Shear 2006, p. 192.

##### Type species.

Martensolasma jocheni Shear, 2006, by monotypy.

##### Diagnosis.

Ortholasmatines lacking a median hood process on the eye tubercle and having a simple pattern of keel cells; scutum magnum present.

##### Maratensolasma jocheni

Shear, 2006

Martensolasma jocheni Shear 2006, p. 193.

###### New record:

24 km north of Xicotepec de Juarez, 1070 m asl, oak forest litter, Puebla, México, collected 17 June 1983 by R. Anderson, male (AMNH).

###### Notes.

This specimen is virtually identical to the holotype and paratype males from Aguascalientes, Aguascalientes, México, despite having been collected about 480 km (300 miles) east-southeast of there. The Aguascalientes type specimens came from a garden, and I speculated that they might have been introduced, but then only considered introductions from outside México. While it is possible (though unlikely) that the species has a natural range of this size, the Aguascalientes specimens could have been brought to that major urban center with garden plants from Puebla. Future collecting may solve the dilemma, and may also produce the presently unknown females.

#### Cladolasma

Suzuki, 1963

Cladolasma Suzuki 1963, p. 40.
Dendrolasma  Shear & Gruber, 1983, p. 51 (in part, only Dendrolasma parvulumSuzuki, 1963; complete pre-1983 references).

##### Type species.

Cladolasma parvula Suzuki, 1963, by monotypy. Shear and Gruber (1983) changed the name of the type species to parvulum to agree in gender with the generic name.

##### Diagnosis.

Ortholasmatines with single lateral hood processes on each side of eye tubercle (two or more such processes in Ortholasma and Trilasma), two-thirds or more length of median hood process; metapeltidium free from abdominal scutum (fused to scutum in Dendrolasma); scute ornamentation of small keel cells only (large or small cells in Dendrolasma); leg femoral microsculpture of broad denticles. Penis with short stylus, two lateral rows of macrosetae.

##### Included species:

In addition to the type, Cladolasma angka (Schwendinger & Gruber 1992).

##### Distribution.

Southern Japan; northern Thailand.

##### Notes.

While the male of Cladolasma angka is unknown, Schwendinger and Gruber (1992) noted that the penis of Cladolasma parvulum is quite different from that of the North American Dendrolasma, with a shorter shaft, compressed glans and short, slender, pointed stylus—opposed to the long, thin shaft, flattened glans and spiral stylus of Dendrolasma (see illustrations of Cladolasma parvulum in Shear and Gruber 1983; penis unknown in Cladolasma angka). Schwendinger and Gruber (1992) pointed out several additional consistent differences between the North American and Asian species placed in Dendrolasma, and suggested the revalidation of Cladolasma for the two known Asian species. However, they described angka under Dendrolasma, and did not formalize the revalidation. The wide geographical separation of the two known species of Cladolasma and the dearth of collecting of soil and litter animals in southeast Asia and southern China almost guarantees the addition of numerous species to this genus in the future.

With regard to Dendrolasma, the two species now included there are quite different from one another. Dendrolasma mirabile Banks 1894 has a scute ornamentation of large cells ranged in rows, while in Dendrolasma dentipalpe Shear and Gruber, 1983, there are  only small cells even smaller than in Cladolasma species. In addition, Dendrolasma dentipalpe has unique secondary sexual modifications in males, including exaggerated cheliceral apophyses and a distomedial tooth on the palpal patella. Discovery of further species could lead to additional splitting of Dendrolasma.

#### Ortholasma

Banks, 1894

Ortholasma Banks 1894, p. 11; Shear and Gruber 1983, p. 14 (in part; with all pre-1983 references); Shear and Gruber 1987, p. 134.

##### Type species.

Ortholasma rugosum Banks 1894, by monotypy.

##### Emended diagnosis.

Ortholasmatines in which the median hood process arises rostrally and is distinctly spoon-shaped, widest past its midlength, and lacks a median, dorsal row of T-shaped tubercles (Trilasma species have parallel-sided median hood processes with a median row of tubercles); two lateral hood processes; males with glands dorsally on the first cheliceromere (glands lacking in Trilasma); male first cheliceromere with or without distal inner tooth; males of some species with palpal femoral and patellar glands (needs verification for all species using scanning electron microscopy); keel cells large, not subdivided into smaller cells (some cells subdivided in Trilasma; sometimes in Dendrolasma).

##### Notes.

Ortholasma species are best distinguished from one another by characters of the dorsal ornamentation, in particular the numbers of lateral T-shaped tubercles on the median hood process and the elevation of the median paired spines of the scute. The proportions of the appendages and their microsculpture are also valuable, but the form of the penis is so similar in all species that it is of little systematic value. The absence of an inner tooth on the basal articles of the chelicerae of males may be synapomorphic for at least some Ortholasma; however, Ortholasma rugosa has a small tooth, and the tooth is not present in all species of Trilasma.

As delimited here, Ortholasma is found almost exclusively in the United States and Canada, though Ortholasma coronadense extends into Baja California, México, on Islas Coronados, offshore and just south of the border with the United States. This species has not been reported from mainland Baja California, but likely occurs there.

##### Key to species of Ortholasma (adapted from Shear and Gruber 1983)

**Table:** 

1.	Large animals, males 4.5 mm or longer, females 6 mm or longer; southern Sierra Nevada in Tulare and Kern Cos., California	Ortholasma colossus sp. n.
–	Smaller animals, males less than 4 mm, females 4.5 mm or less	2
2.	Leg femora banded, body with median light stripe; northern California north to Vancouver Island	Ortholasma pictipes Banks
–	Leg femora not banded, body uniform in color	3
3.	Leg femora without false articulations; San Francisco Bay area	Ortholasma rugosum Banks
–	At least second femora with false articulations	4
4.	All femora with false articulations; San Francisco Bay area south to Los Angeles, Sierra Nevada foothills, caves in higher Sierras	Ortholasma levipes Shear & Gruber
–	False articulations only in femora 2 and 4; southern California and Coronado Islands	Ortholasma coronadense Cockerell

##### Ortholasma colossus sp. n.

urn:lsid:zoobank.org:act:78E03B21-192C-47C3-B964-C179BC06EF60

**Figure 1:**
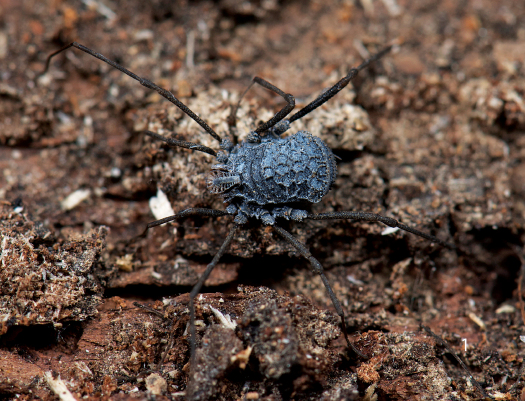
Ortholasma colossus sp. n., live female specimen in habitat. Photo courtesy of Marshal Hedin.

Figs 1 8 10 20 30, 31 

###### Types.

Male holotype and two female paratypes collected in Bear Den Cave, Sequoia National Park, Tulare Co., California, 1 May 2004, by J. Krejcaet al. deposited in California Academy of Sciences (CAS); male and female paratypes from same locality, collected 17–18 July 2003 by J. Krejca et al. deposited in Field Museum of Natural History (FMNH).

###### Diagnosis.

The notably larger size of the females distinguishes this species of Ortholasma from all others.

###### Etymology.

The species name, a Latin noun in apposition, refers to a gigantic statue, or by implication, anything outlandishly large for its type.

###### Description.

**Figures 2, 3.**
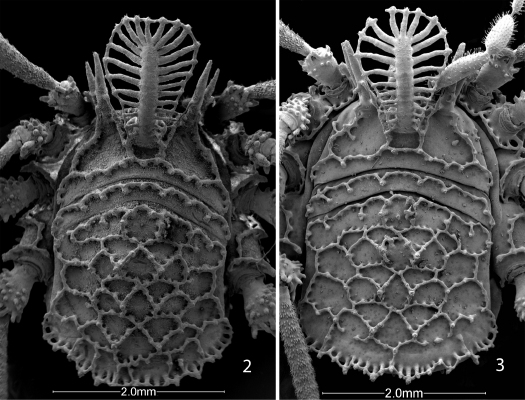
Scanning electron micrographs of Ortholasma colossus sp. n., dorsal views. **2** male **3** female.

Male holotype: total length, 4.5, width, 2.25. Color uniform blackish brown, legs somewhat lighter brown. Carapace arcuate, about twice as wide as long, with complete lateral and posterior submarginal keels; median keels connect eye tubercle and innermost lateral hood process, lateral keels also arising on innermost lateral hood process. Two acute lateral hood processes each about half as long as median hood process. Circumocular keels absent, only subocular portion present. Median hood process arising dorsally on eye tubercle, length 1.6, width 1.0; bearing 24 lateral T-shaped tubercles, all connected. Metapeltidium free, complete keel along anterior margin. Scute 2.6 long, 2.25 wide. Pattern of scute keels typical, paired median scute spines low, scarcely standing above level of keels; posterior marginal keel with complete fenestrations (Fig. 2).

**Figures 4–9.**
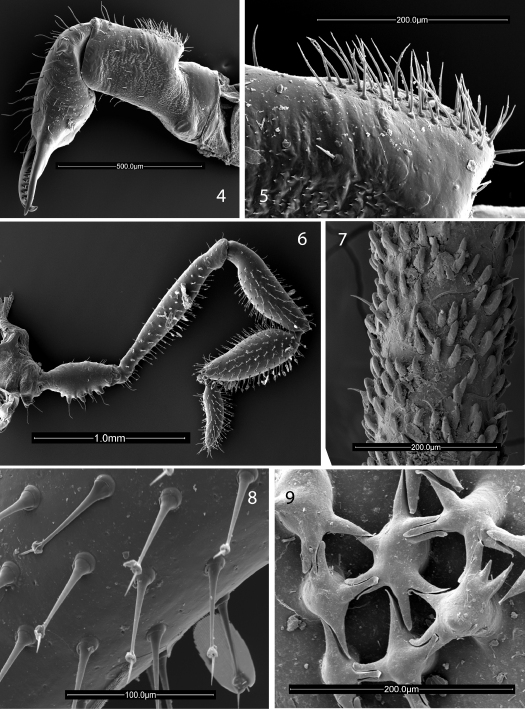
Scanning electron micrographs. **4-8** Ortholasma colossus sp. n. **4** right chelicerae of male, mesal view **5** glandular area of basal cheliceral article of male, mesal view **6** right palpus of male, mesal view **7** sculpture of second femur of female **8** kugelhaare of female palpus **9** Trilasma trispinosum sp. n., branched tubercles forming small keel cells of scute.

**Figures 10–19.**
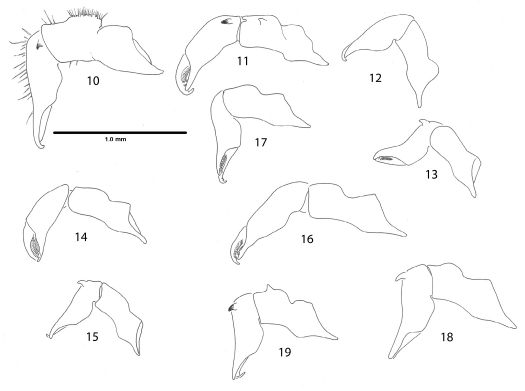
Right chelicerae, mesal views. **10** Ortholasma colossus sp. n., male **11** Trilasma ranchonuevo sp. n., male **12** Trilasma trispinosum sp. n., female **13** Trilasma trispinosum sp. n., male **14** Trilasma tempestado sp. n., female **15** Trilasma tempestado sp. n., male **16** Trilasma petersprousei sp. n., female **17** Trilasma chipinquensis sp. n., female **18** Trilasma hidalgo sp. n., male **19** Trilasma tropicum sp. n., male. All drawings to the same scale.

**Figures 20–29.**
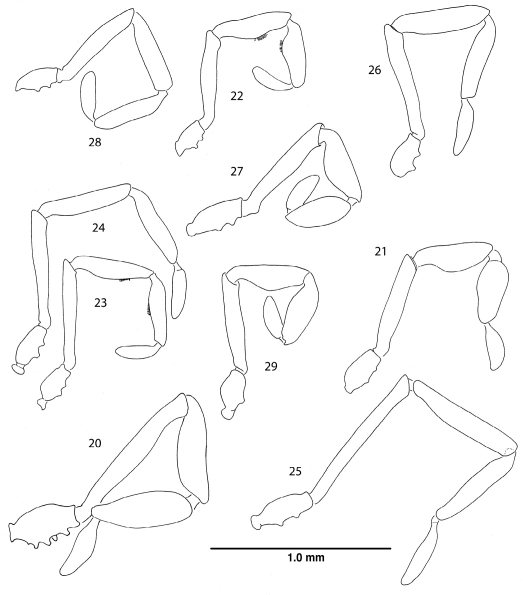
Right palpi, lateral views. **20** Ortholasma colossus sp. n., male **21** Trilasma ranchonuevo sp. n., male **22** Trilasma trispinosum sp. n., male **23** Trilasma tempestado sp. n., male **24** Trilasma tempestado sp. n., female **25** Trilasma petersprousei sp. n., female **26** Trilasma chipinquensis sp. n., female **27** Trilasma hidalgo sp. n., male **28** Trilasma tropicum sp. n., female **29** Trilasma tropicum sp. n., male. All drawings to the same scale.

Chelicerae (Figs 4, 5, 10) with basal article 0.9 long, 0.25 wide, prominent epigamic gland with dense vestiture of small, fine setae (Fig. 5), otherwise sparsely setose; second article 0.75 long, 0.21 wide, with small, curved mediobasal tooth. Palpus (Figs 6, 20) with dense vestiture of clavate setae; trochanter with several ventral seta-tipped tubercles; tibia swollen; dimensions given in Table 1. Legs in order of length, 2 (11.95), 4 (9.7), 3 (6.3), 1 (5.8); metatarsus 2 with 8 false articulations, otherwise false articulations lacking; tarsi 1–4 with 10, 11, 10, 11 articles respectively. Lengths of leg segments given in Table 1. Leg femora with typical ornamentation (Fig. 7).

**Figures 30–41.**
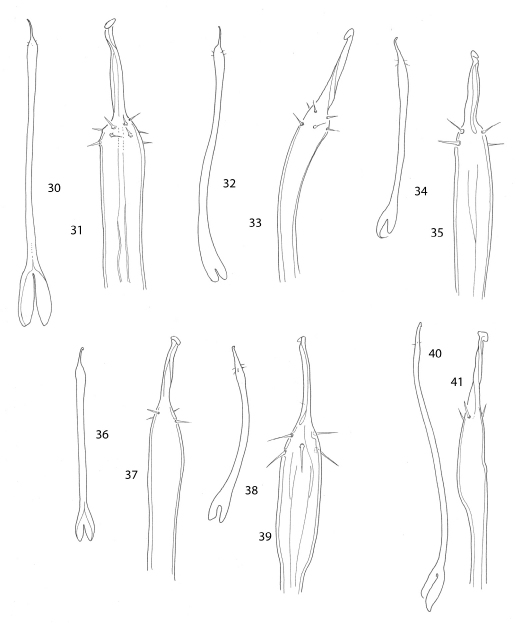
Penes. **30**, **31** Ortholasma colossus **30** dorsal view **31** glans **32**, **33** Trilasma ranchonuevo sp. n. **32** dorsal view **33** glans **34**, **35** Trilasma trispinosum sp. n. **34** dorsal view **35** glans **36**, **37** Trilasma tempestado sp. n. **36** dorsal view **37** glans **38**, **39** Trilasma tropicum sp. n. **38** dorsal view **39** glans **40**, **41** Trilasma hidalgo sp. n. **40** lateral view **41** glans. All drawings to the same scale; penis of Ortholasma colossus is 1 mm long.

Genital operculum broadly rounded, marginate, suture faintly indicated. Penis (Fig. 30) 1.0 long, typically thin; glans (Fig. 31) sinuate with hooked tip, subtended by small setae.

Female paratype: total length, 6.6, width, 4.2. Nonsexual characters as in male (Fig. 3); median hood process 1.7 long, 0.9 wide. Scute 4.5 long, 4.2 wide. Chelicera without gland on basal article, distal article lacking tooth, basal article 1.05 long, 0.26 wide; distal article 0.90 long, 0.24 wide. Palpal tibia not swollen, dimensions given in Table 2. Legs in order of length, 2 (10.6), 4 (7.34), 3 (5.7), 1 (4.87); metatarsus 2 with 6 false articulations; tarsi 1–4 with 7, 8, 9, 11 articles respectively. Lengths of leg segments given in Table 2. Genital operculum broadly rounded, marginate, without suture. Ovipositor typical.

**Table 1.** Appendage article measurements (mm) of Ortholasma colossus male.

	Femur	Patella	Tibia	Metatarsus	Tarsus
Palpus	1.10	0.76	0.70	-	0.52
Leg 1	1.90	0.60	1.10	1.10	1.10
Leg 2	4.00	0.75	3.50	2.50	1.20
Leg 3	1.90	0.40	2.00	0.90	1.10
Leg 4	3.20	1.00	3.00	1.30	1.20

###### Distribution.

All specimens deposited in CAS unless otherwise noted. CALIFORNIA: Fresno Co., 3 mi. south of Trimmer, 4 June 1967, under rhyolite in oak forest, T. Briggs, female (AMNH). Tulare Co., Johnsondale, Kern River, 4 July 1956, V. Roth, W. Gertsch, female (AMNH); Lost Soldier’s Cave, December 1977, A. Grubbs, female (AMNH); 21 July 2003, J. Krejcaet al., female, 23 July 2003, molt fragment, 9 November 2003, female; Lightening Cave, 30 June 1952, A. Lange, female (used for SEM; AMNH); Sequoia National Park, Ash Mtn., 26 April 1951, E. Schlinger, male (AMNH); Paradise Cave, 30 April 2004, J. Krejca et al., male, juveniles; Lange Cave, 6 May 2004, J. Krejca et al., male, 2 females, 6 May 2004, male, female, 7 May 2004, late instar juvenile; Carmoe Crevice, 5 July 2003, J. Krejcaet al., male; May’s Cave, 16 May 2004, J. Krejca et al., female, early juvenile; Hidden Cave, 15 November 2003, J. Krejca, V. Loftin, male; Highway 245, 14 mi. north of Woodlake, near Cottonwood Creek and Rattlesnake Creek confluence, 26 March 2009, M. Hedin et al., female (pictured alive and in the field in Fig. 1).

**Table 2.** Appendage article measurements (mm) of Ortholasma colossus female.

	Femur	Patella	Tibia	Metatarsus	Tarsus
Palpus	1.10	0.90	0.70	-	0.46
Leg 1	1.50	0.52	1.20	1.00	1.10
Leg 2	3.50	0.80	2.60	2.10	1.60
Leg 3	1.80	0.52	1.52	0.90	0.96
Leg 4	2.20	0.80	2.20	1.10	1.04

###### Notes.

The preponderance of cave records is probably misleading; the species shows no signs of troglomorphosis and specimens from caves are nearly identical to the few surface-collected specimens. The cool, moist environment of caves probably attracts individuals (troglophily). Caves have been much more thoroughly collected than surface habitats, and in this case most of the records come from a biological survey of the caves of Sequoia and Kings Canyon National Parks. The caves mentioned in the Distribution section are located on a map in Shear and Shelley (2008: Fig. 9). The Fresno Co. record may be questionable. The label places Trimmer in Kern Co., but according to all sources, it is an unincorporated community in Fresno Co., on the north shore of the Pine Flat Reservoir (shown on some maps as Isabella Reservoir). The elevation in the vicinity is less than 610 m (2000’) asl, while no other records of the species but one (near Woodlake) come from below 2100 m (about 7000’) asl. If accurate, this record suggests a wider distribution for Ortholasma colossus sp. n.

The size of the species, at least 50% longer than the next largest, is striking when seen side-by-side with congeners; only Ortholasma rugosum approaches it with some 4.5-mm-long males. However, most males of Ortholasma colossus sp. n. are longer than 4.5 mm, and the largest females of Ortholasma rugosum are only 4.8 mm long, compared to the usual 6–7 mm in colossus sp. n.

#### Trilasma

Goodnight & Goodnight, 1942

Trilasma Goodnight and Goodnight 1942, p. 7.
Ortholasma Shear and Gruber 1983, p. 14 (in part; only the species bolivari and sbordonii; see for complete references before 1983).
Ruaxphilos Goodnight & Goodnight 1945a, p. 299. First synonymized with Ortholasma by Shear and Gruber (1983).

##### Type species.

 Trilasma bolivari Goodnight & Goodnight, 1942, by monotypy. Of Ruaxphilos, Ruaxphilos petrunkevitchou Goodnight & Goodnight, 1945, by monotypy.

##### Emended diagnosis.

Ortholasmatines in which the median hood process arises dorsally, projects anteriorly in a shallow curve, and is parallel-sided or nearly so, with a dorsal row or rows of tubercles (spoon-shaped and lacking dorsal tubercles in Ortholasma); two or three lateral hood processes; males without cheliceral glands (cheliceral glands present in Ortholasma); male first cheliceromere with distal inner tooth, dorsal tooth or sharp tubercle present or absent, curved mediobasal tooth on second cheliceromere (not verified for species known only from females); palpal patella with only two seta-bearing tubercles (Ortholasma with several); large keel cells separated by transverse rows of much smaller keel cells (Ortholasma lacks rows of smaller cells; Dendrolasma has either large or small cells but not both).

##### Notes.

Trilasma consists of seven species extending from Nuevo León, México, in the north to Honduras in the south. Not much ecological information is available. One species, sbordonii, is troglomorphic, but while other cave collections exist, none of the other species seems especially adapted for life underground (although both petersprousei sp. n. and tempestado sp. n. have unusually long, thin legs and palpi) and probably are at most troglophilic. Surface collections are from cloud forest litter, and from under the bark of pines. Except for chipinquensis, collected at an altitude of about 1525 m (5000’), all species come from altitudes ranging from 2100–3050 m (7000–10000’) asl.

**Figures 42, 43.**
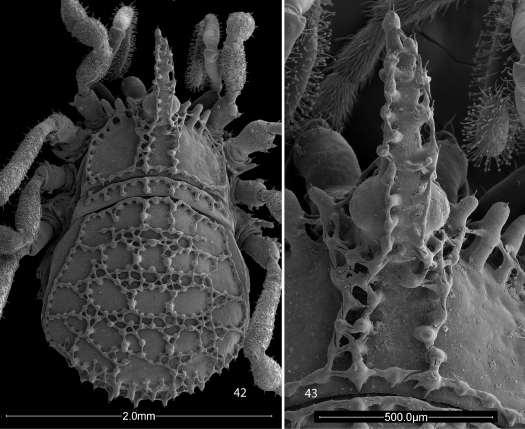
Scanning electron micrographs of female Trilasma trispinosum sp. n. **42** body, dorsal view **43** hood, dorsal view.

**Figures 44, 45.**
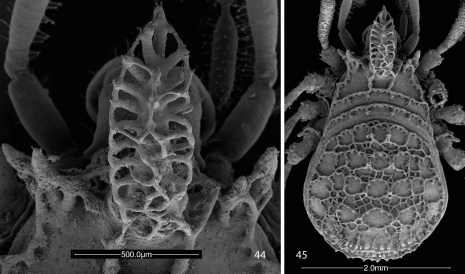
Scanning electron micrographs of female Trilasma tempestado sp. n. **44** hood, dorsal view **45** body, dorsal view.

Trilasma species are immediately separable from Ortholasma species due to the presence of areas of small keel cells on the abdominal scutum (compare Figs 42, 45, and 54 with 1, 2). It appears that the small cell groups are at the anterior borders of the scute areas because in some species the first group is to be found at the very anterior margin of the scute (of area 1) and succeeding groups appear anterior to the paired median spines of the succeeding areas. The maximum number of groups is thus five, but the anteriormost is absent in some species, and the posteriormost is generally broken up into two groups on either side of the midline and consists of just two or three small cells on each side, or may be absent as well. The small cell groups of areas 2, 3 and 4 are the most marked and are successively wider, with the group of area 4 occupying about two-thirds the width of the scute and consisting of up to 20 small cells. In addition, Trilasma species have dorsal tubercles on the median hood process of the eye tubercle; these may be few (trispinosum sp. n., Fig. 43) or very numerous (tempestado sp. n., Fig. 44) and bearing branches that connect complexly with each other and with the usual lateral tubercles. Instead of the typically spatulate dorsal hood processes of Ortholasma species, Trilasma species have dorsal hood processes with nearly parallel sides that are obviously longer and narrower than in the former genus, reaching an extreme in the very narrow hood of Trilasma trispinosum sp. n. (Fig. 43). In several species the process tapers apically to a point.

Trilasma species are on average significantly smaller than Ortholasma species, ranging from a minimum of 2.1 mm in length to just 3.4 mm, including the hood. As in other members of the subfamily, the order of legs in length is 2, 4, 3, 1. In some species false articulations are present in at least some femora and metatarsi, but sometimes only in males. The detailed ornamentation of the leg femora, of taxonomic value in Ortholasma, because it differs between species, is quite uniform in Trilasma, consisting of numerous, short, club-shaped setae, among which are dispersed a few blunt, rod-shaped setae on elevated sockets. This character was studied for all species using scanning electron microscopy. The short, club-shaped setae are clearly seen to be hollow in some of the photomicrographs, but there appear not to be any pores communicating with the outside. In species of both Ortholasma and Dendrolasma, the longer setae on elevated sockets are acute and tapered, not blunt and rod-shaped.

Sexual dimorphism in Trilasma species is more pronounced than in Ortholasma species. Males are significantly smaller than females, with relatively longer legs, especially legs 2 and 4, which may also have increased numbers of tarsal segments, and in some species the males have false articulations in the fourth leg femora that are not present in the females. In addition to the basal, median tooth on the second cheliceral segment and the glands in the patellae and tibiae of the palpi, males of Trilasma species may have somewhat reduced dorsal ornamentation when compared to females, expressed in fewer small cells and lower paired tubercles of the dorsal areas. This dimorphism is especially notable at the posterior margin of the scute, where in females there is an obvious “post and rail fence” with long “posts” (Shear and Gruber, 1983); in males of the same species, this feature may be hardly noticeable.

The following characters seem to be useful in defining and separating the species of Trilasma: the relative length and robustness of the legs, the presence or absence and the number (if present) of false articulations in the femora and metatarsi of the legs (this may be sexually dimorphic in some species), the range of numbers of tarsal articles (difficult to evaluate because of small sample size; the counts given should be taken as variable by one or two articles in either direction), the relative prominence of the ocular keels, the patterns of small keel cells on the scute, the height of the paired area tubercles, and the shape and size of the dorsal hood process. In addition, there are autapomorphies of single species, such as the very narrow dorsal hood process and three lateral hood processes in Trilasma trispinosum sp. n.

While I am still certain that Ruaxphilos is a synonym of Trilasma, it is no longer clear that Ruaxphilos petrunkevitchou Goodnight & Goodnight, 1945 is a synonym of Trilasma bolivari, as placed by Shear and Gruber (1983). The type specimen of the former species came from Veracruz, whereas the latter is known from several localities in the transverse volcanic belt of central México.

##### Key to species of Trilasma

**Table:** 

1.	Troglomorphic facies, depigmented animals with reduced eyes and elongate legs and palpi; caves in Tamaulipas	Trilasma sbordonii (Šilhavý)
–	Medium tan or dark brown to black animals with normal eyes	2
2.	Two lateral hood processes	4
–	Three lateral hood processes	3
3.	Femora 2 and 4 of males each with a false articulations (females unknown); median hood process normally broad, with 5 or 6 dorsal tubercles; Tamaulipas	Trilasma ranchonuevo sp. n.
–	All femora of males with false articulations; median hood process extremely narrow, with only 1 or 2 dorsal tubercles; Veracruz	Trilasma trispinosum sp. n.
4.	Femur of second leg longer than or equal to scute length	5
–	Femur of second leg shorter than scute	6
5.	Fourth femur with two false articulations; second femur about as long as scute; Nuevo Léon	Trilasma tempestado sp. n.
–	All femora without false articulations; second femur longer than scute; San Luis Potosí	Trilasma petersprousei sp. n.
6.	Fourth femur with a basal false articulation; Nuevo Léon	Trilasma chipinquensis sp. n.
–	All femora lacking false articulations	7
7.	Second metatarsus with two or three false articulations; Distrito Federal, Puebla and adjacent states	Trilasma bolivari Goodnight & Goodnight
–	Second metatarsus without false articulations	8
8.	Males about 3.0 mm long; about 15 dorsal tubercles on median hood process; Hidalgo, México	Trilasma hidalgo sp. n.
–	Males about 2.5 mm long; about 8 dorsal tubercles on median hood process; Honduras	Trilasma tropicum sp. n.

##### Trilasma ranchonuevo sp. n.

urn:lsid:zoobank.org:act:7BCAB326-0BF9-414A-8E16-BC5773077567

Figs 11 21 32, 33 47 

###### Type.

Male holotype (TMM) from Rancho Nuevo, Tamaulipas, México, collected 10 April 1982 by Terri Treacy.

###### Diagnosis.

Like Trilasma trispinosum sp. n., ranchonuevo sp. n. has three lateral hood processes, but the median hood process of ranchonuevo sp. n. is much broader and has more dorsal tubercles. There are more small keel cells on the scute of trispinosum sp. n. than in ranchonuevo sp. n.The male of ranchonuevo sp. n. has a single false articulation in each of the second and fourth leg femora; such are present in all legs of trispinosum sp. n. males. Tarsal counts of the single available specimen are 3, 4, 4, 4, the lowest for any Trilasma.

###### Etymology.

The species epithet, a noun in apposition, refers to the type locality.

###### Description.

Male holotype: total length, 2.7, width, 1.6. Color pale tan to yellowish brown. Carapace arcuate, about 1.5X as wide as long, with complete lateral and posterior submarginal keels; pair of median keels connecting eye tubercle and innermost lateral hood process, lateral keels arising both on innermost and middle lateral hood processes. Three blunt lateral hood processes each about one-half as long as median hood process. Circumocular keels suppressed, subocular portion vaguely indicated, eyes relatively large, bulging. Median hood process arising dorsally on eye tubercle, with nearly parallel sides, then converging distally, length 0.9, width 0.3; median keels of carapace continuing as rows of lateral tubercles on median hood process, about 18–20 lateral tubercles, linearly connected; 5 or 6 dorsal tubercles present, connected linearly to one another but not obviously to lateral tubercles. Metapeltidium free, complete keel along anterior margin, 6 tubercles posterior to keel, connected to it by single branch each. Scute 1.6 long, 1.6 wide. All keels relatively low. Small keel cells present only on areas 2–4, as single transverse rows of 6–8 cells. Paired median scute spines prominent, on areas 4, 5 larger than adjacent keel tubercles (Fig. 47).

Chelicerae (Fig. 11) with basal article 0.62 long, 0.22 wide, sparsely setose, with small, median distal tooth; second article 0.62 long, 0.20 wide, with dark, median basal tooth. Palpus (Fig. 21) with dense vestiture of clavate setae; patella and tibia swollen, but glands not marked by patches of small setae, trochanter with ventral seta-tipped tubercles very low, almost obselete; dimensions given in Table 3. Legs in order of length, 2 (6.66), 4 (4.74), 3 (3.12), 1 (3.0); metatarsus 2 with 2 false articulations, femora 2, 4 with single basal false articulation; tarsi 1–4 with 3, 4, 4, 4 articles respectively. Lengths of leg segments given in Table 3. Length/width ratios of femora, in order: 3.64, 8.33, 3.46, 7.0. Leg femora with typical ornamentation.

Genital operculum broadly rounded, marginate, notched. Penis (Figs 32, 33) typical of genus.

**Table 3.** Appendage article measurements of Trilasma ranchonuevo sp. n. male.

	Femur	Patella	Tibia	Metatarsus	Tarsus
Palpus	0.70	0.50	0.50	-	0.31
Leg 1	0.80	0.40	0.60	0.80	0.40
Leg 2	1.50	0.60	1.20	1.94	1.60
Leg 3	0.90	0.44	0.66	0.72	0.40
Leg 4	1.40	0.52	1.00	1.00	0.82

###### Notes.

The male holotype of Trilasma ranchonuevo sp. n. is significantly larger than the male paratype of Trilasma trispinosum sp. n. and has longer legs. The pattern of small cells on the scute is quite different, and the median hood process is much broader in relation to its width, with many more dorsal tubercles. On the left side of the body of the holotype, the innermost lateral hood process is irregularly developed, suggesting that the innermost process is the “extra” one and that its presence or absence might be subject to variation.

Rancho Nuevo is in the Sierra Nevada Oriental in western Tamaulipas, near the Nuevo Léon border and about 22 miles northwest of Ciudad Victoria. Coordinates: 23°51'50.40N; 99°27'07.43W, elevation 8600’ (2650 m).While the region around Rancho Nuevo is famous for its caves, this specimen was collected on the surface and shows no signs of troglobiosis.

##### Trilasma trispinosum sp. n.

urn:lsid:zoobank.org:act:F39EF9AA-DD3B-48F2-8C8E-B0EC3E17BF6D

Figs 9 12, 13 22 34, 35 42, 43 

###### Types.

Female holotype, male and female paratypes (TMM) from Puerto del Aire, Veracruz, México, collected 6 January 1966 by J. Richter, in cloud forest oak litter.

###### Diagnosis.

Differing from all other Trilasma except ranchonuevo sp. n.in the three, rather than two, lateral hood processes of the carapace, but distinct from ranchonuevo sp. n. in the extremely narrow median hood process, with only a few dorsal tubercles.

###### Etymology.

The species epithet refers to the three lateral hood processes.

###### Description.

Female holotype: total length, 2.6, width, 1.7. Color uniform chestnut brown, legs somewhat lighter brown, proximal parts of femora whitish yellow. Carapace arcuate, about 1.5X as wide as long, with complete lateral and posterior submarginal keels; pair of median keels connect eye tubercle and innermost lateral hood process, lateral keels also arising on innermost lateral hood process. Three blunt lateral hood processes each about one-fourth as long as median hood process. Circumocular keels absent, subocular portion vaguely indicated, eyes relatively large, bulging. Median hood process arising dorsally on eye tubercle, narrow, length 0.9, width 0.11; median keels of carapace continue as rows of reduced lateral tubercles on median hood process, about 18–20 lateral tubercles, linearly connected; only 2 or 3 dorsal tubercles present. Metapeltidium free, complete keel along anterior margin, 6 tubercles posterior to keel, connected to it by single branch each. Scute 1.5 long, 1.7 wide. All keels relatively low. Small keel cells of scute area 1 absent, small keel cell rows progressively wider posteriorly, widest on area 4; small cells of area 5 in two groups either side of midline. Paired median scute spines small, on areas 4, 5 no larger than adjacent keel tubercles (Figs 42, 43).

Chelicerae (Fig. 12) with basal article 0.65 long, 0.18 wide, sparsely setose; second article 0.52 long, 0.18 wide. Palpus with dense vestiture of clavate setae; trochanter with ventral seta-tipped tubercles; dimensions given in Table 4. Legs in order of length, 2 (5.45), 4 (4.99), 3 (3.10), 1 (3.05); metatarsi without false articulations, femur 4 with single basal false articulation; tarsi 1–4 with 4, 5, 4, 5 articles respectively. Lengths of leg segments given in Table 4. Length/width ratios of femora, in order: 3.5, 7.0, 3.75, 5.25. Leg femora with typical ornamentation.

Genital operculum broadly rounded, marginate, with suture. Ovipositor typical of subfamily.

Male paratype: total length, 2.3, width, 1.2. Nonsexual characters as in female, but dorsal ornament reduced, obscured in paratype by secretion; median hood process 0.79 long, 0.2 wide. Scute 1.2 long, 1.2 wide. Chelicera (Fig. 13) without gland on basal article, second article with forward-projecting tooth, basal article 0.60 long, 0.20 wide; distal article 0.51 long, 0.15 wide. Palpal patella and tibia swollen, small ventral glandular areas indicated by patches of fine setae (Fig. 22), dimensions of palpus given in Table 5. Legs in order of length, 2 (5.76), 4 (4.96), 3 (3.32), 1 (3.22); all femora with single, basal false articulation, metatarsi without false articulations; tarsi 1–4 with 4, 5, 4, 5 articles respectively. Lengths of leg segments given in Table 5. Length/width ratios of femora, in order: 3.9, 9.8, 4.0, 6.1. Genital operculum broadly rounded, marginate, with two small, lateral notches. Penis typical (Figs 34, 35).

**Table 4.** Appendage article measurements of Trilasma trispinosum sp. n. female.

	Femur	Patella	Tibia	Metatarsus	Tarsus
Palpus	0.60	0.57	0.50	-	0.25
Leg 1	0.70	0.40	0.50	0.80	0.65
Leg 2	1.05	0.55	0.70	1.60	1.55
Leg 3	0.75	0.40	0.50	0.80	0.65
Leg 4	1.05	0.40	0.74	1.00	1.80

**Table 5.** Appendage article measurements of Trilasma trispinosum sp. n. male.

	Femur	Patella	Tibia	Metatarsus	Tarsus
Palpus	0.65	0.51	0.49	-	0.30
Leg 1	0.78	0.40	0.50	0.80	0.74
Leg 2	1.18	0.44	0.74	1.70	1.70
Leg 3	0.80	0.38	0.60	0.74	0.80
Leg 4	1.10	0.46	0.80	1.30	1.30

###### Notes.

This species is the most distinctive of all Trilasma species due to the very short and narrow median hood process, which has only one or two dorsal armed tubercles. The female holotype (Figs 42, 43) is anomalous in that the keel extending from the eye tubercle to the posterior margin of the carapace is doubled on the left side. The pattern of keel cells is very distinct in the female holotype and paratype, but the male paratype has a much-reduced ornament. However, this is uncertain because of the thick secretion covering the dorsum and median hood process in the single available specimen; this material could not be cleaned off using ultrasonics, or methanol as a solvent. Notable also is the presence in the male of basal false articulations in all the femora. In the female, only the fourth femur has a basal false articulation. The presence of palpal glands in the male palpal patellae and tibiae is indicated by the patch of fine setae on the ventral side, and in still-adhering secretion on the tibia.

Puerto del Aire is a small village west of the larger city of Acultzingo, Veracruz, and southeast of Morelos Canadá, Puebla, virtually at the Puebla-Veracruz border (18°42'13.5N; 97°21'29.6W). The elevation is about 2556 m (7500 ft.) asl.

##### Trilasma tempestado sp. n.

urn:lsid:zoobank.org:act:2755568D-A57D-4B19-80BF-62EE1547B1DC

Figs 14, 15 23, 24 36, 37 44, 45 48 

###### Types.

Holotype female and male paratype (TMM) from Cueva de Polvo Tempestado, 1–2 km south of San Josecito, Nuevo Léon, México, collected 1 March 1989 by George Veni and Allan Cobb; female paratype (TMM) from Sótano de las Tres Ventanas, Purifacición area, Cuauhtémoc, Nuevo Léon, México, collected 29 November 1981 by Paul Fambro; male paratype (TMM) from Pozo de las Pantaletas, Santa Marta de Arriba, 20 km southeast of Zaragoza (UTM 433010/2656459), collected 26 November 1999 by Peter Sprouse. See Notes, below, for a detailed discussion of these localities.

###### Diagnosis.

A long-legged, pale species most similar to Trilasma petersprousei sp. n., which is about half again as large. Trilasma tempestado sp. n.has the second femur about the same length as the scute, and has two false articulations in the fourth femora of both males and females, while petersprousei sp. n. lacks femoral false articulations. With the male paratype only 2.1 mm in length, this species is the smallest known Trilasma, about the same size as Martensolasma jocheni, the smallest known ortholasmatine.

###### Etymology.

The species epithet is a noun in apposition, referring to the type locality.

###### Description.

Female holotype: total length, 2.6, width, 1.5. Color pale tan to yellowish brown. Carapace arcuate, about 1.5X as wide as long, with complete lateral and posterior submarginal keels; pair of median keels connecting eye tubercle and innermost lateral hood process, lateral keels arising both on innermost and middle lateral hood processes. Two blunt lateral hood processes each about one-third or less as long as median hood process. Circumocular keels suppressed, but subocular portion visible, eyes relatively small. Median hood process arising dorsally on eye tubercle, with nearly parallel sides, then converging distally, widest at about midlength, length 0.90, width 0.40; median keels of carapace continuing as rows of lateral tubercles on median hood process, about 20 lateral tubercles, linearly connected; 11–12 dorsal tubercles present, connected complexly to one another and to lateral tubercles. Metapeltidium free, complete keel along anterior margin, 4 tubercles posterior to keel, connected to it by single branch each. Scute 1.6 long, 1.5 wide. All keels relatively high. Small keel cells present on areas 1–5; area 1 with 3–4 small cells in midline, area 2 with about 10 small cells in transverse row less than ½ width of scute, area 3 with about 16 small cells in midline, area 4 with 20–23 small cells in transverse row about 2/3 width of scute, area 5 with two paramedian groups of 3–4 small cells. Paired median scute spines relatively prominent, on all areas distinctly larger than adjacent keel tubercles (Figs 44, 45).

Chelicerae (Fig. 14) with basal article 0.60 long, 0.20 wide, sparsely setose; second article 0.56 long, 0.18 wide. Palpus (Fig. 24) slender, with dense vestiture of clavate setae; trochanter with two prominent seta-bearing ventral tubercles; dimensions given in Table 6. Legs in order of length, 2 (8.80), 4 (6.12), 3 (4.48), 1 (4.32); metatarsus 2 with 3 false articulations, femora 4 with 3 false articulations; tarsi 1–4 with 5, 6, 7, 8 articles respectively. Lengths of leg segments given in Table 6. Length/width ratios of femora, in order: 7.33, 16.67, 8.0, 12.14. Leg femora with typical ornamentation.

Genital operculum broadly rounded, separated from sternite by suture. Ovipositor typical of genus.

Male paratype: total length, 2.1, width, 1.3. Color uniform light chestnut brown. Nonsexual characters as in female (see Figs 44, 45, 48 and description, above), but dorsal ornament somewhat reduced; median hood process 0.70 long, 0.25 wide. Scute 1.3 long, 1.3 wide. Chelicera (Fig. 15) without gland on basal article, second article with strong, anteriodorsal, slightly hooked, conical protuberance, basal article 0.50 long, 0.17 wide; distal article 0.52 long, 0.16 wide. Palpal patellae and tibiae swollen, epigamic glands present and marked by patches of fine setae (Fig. 23), dimensions of palpus given in Table 7. Legs in order of length, 2 (8.68), 4 (6.00), 3 (4.48), 1 (4.26); metatarsus 2 with 2 false articulations, femur 4 with 3 false articulations; tarsi 1–4 with 5, 5, 6, 6 articles respectively. Lengths of leg segments given in Table 7. Length/width ratios of femora, in order: 7.43, 20.0, 6.89, 10.5. Genital operculum broadly rounded, marginate, with two small, lateral notches. Penis typical (Figs 36, 37).

**Table 6.** Appendage article measurements of Trilasma tempestado sp. n. female.

	Femur	Patella	Tibia	Metatarsus	Tarsus
Palpus	0.80	0.65	0.56	-	0.34
Leg 1	1.10	0.40	0.70	1.12	1.00
Leg 2	2.00	0.60	1.50	2.90	1.80
Leg 3	1.12	0.40	0.80	1.16	1.00
Leg 4	1.70	0.40	1.28	1.50	1.24

**Table 7.** Appendage article measurements of Trilasma tempestado sp. n. male.

	Femur	Patella	Tibia	Metatarsus	Tarsus
Palpus	0.72	0.60	0.46	-	0.30
Leg 1	0.90	0.46	0.64	0.90	0.90
Leg 2	1.50	0.56	1.08	2.00	1.30
Leg 3	0.84	0.50	0.66	0.80	0.96
Leg 4	1.40	0.56	1.28	1.00	1.10

###### Notes.

Like Trilasma petersprousei sp. n., this species is pale and long-legged, yet does not appear to be a troglobiont. It occurs in at least two distinct karst areas. Cueva de Polvo Tempestado is located 1–2 km south of the village of San Josecito (23°58'12.68N; 99°54'21.18W, elev. ca. 2300m (7570’)). This is a small, vertical cave, one of several in the area. Sótano de las Tres Ventanas is a pit in the well-known Río Purificación karst region located in Nuevo Léon and Tamaulipas between Zaragoza, NL, and Ciudad Victoria, Tamps. The coordinates are 23°53’30.583N; 99°28’20.445W, at an elevation of 2210 m asl. The entrance pit drops 35 m to a rubble pile, which slopes down to a plug at -45 meters. Temperature measured at this point was 8.6° C during exploration on 29 November 1981 (P. Sprouse, pers. comm.) Sistema Purificación, located in this area, is one of the deepest and longest cave systems in México. Pozo de las Pantaletas is located near the village of Santa Marta at coordinates of 23°51’40.16N; 99°41’23.982W and an elevation of 2800 m asl. This is a vertical cave with multiple rope drops leading to a depth of 140 m. All of these records are rather tightly clustered in a mountainous region of Nuevo Léon that makes an easterly salient into Tamaulipas 35–45 km northwest of Ciudad Victoria. It would be no surprise to see specimens from the adjoining part of Tamaulipas.

##### Trilasma petersprousei sp. n.

urn:lsid:zoobank.org:act:A8DA8437-E2B4-4F4E-8434-05EEAFAC56BC

Figs 16 25 46 

###### Types.

Female holotype (TMM) from Hoya de las Guaguas, 10 km south of Aquismón, San Luis Potosí, México, collected 29 August 1986 by Peter Sprouse.

###### Diagnosis.

 Trilasma petersprousei sp. n. is a long-legged species, with long, thin pedipalps. The high tarsal count of the holotype (6, 12, 6, 8) and the presence of 12 false articulations in the second metatarsi also distinguish it from other species.

**Figures 46, 47.**
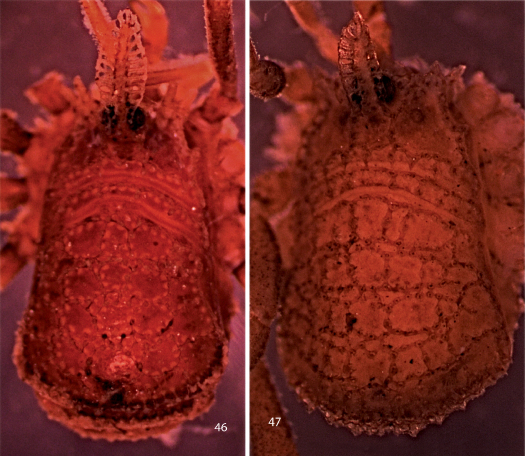
Photographs of bodies, dorsal views. **46** male Trilasma petersprousei sp. n. **47** female Trilasma ranchoneuvo sp. n.

**Figures 48, 49.**
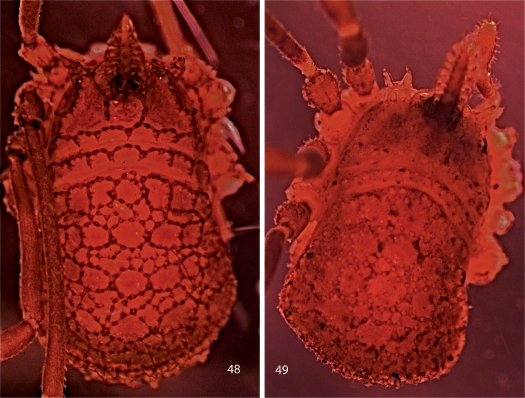
Photographs of bodies, dorsal views. **48** male Trilasma tempestado sp. n. **49** male Trilasma tropicum sp. n.

###### Etymology.

The species is named for the collector, Peter Sprouse of Zara Environmental, LLC, a noted explorer of Mexican caves who has collected many new species of troglobionts and troglophiles.

###### Description.

Female holotype: total length, 3.4, width, 1.9. Color pale tan to yellowish brown. Carapace arcuate, about 1.5X as wide as long, with complete lateral and posterior submarginal keels; pair of median keels connecting eye tubercle and innermost lateral hood process, lateral keels arising both on innermost and middle lateral hood processes. Two blunt lateral hood processes each about one-half as long as median hood process. Circumocular keels suppressed, but subocular portion obvious, eyes relatively large, bulging. Median hood process arising dorsally on eye tubercle, with nearly parallel sides, then converging distally, widest slightly beyond midlength, length 1.10, width 0.45; median keels of carapace continuing as rows of lateral tubercles on median hood process, about 20 lateral tubercles, linearly connected; about 15 dorsal tubercles present, connected complexly to one another and to lateral tubercles. Metapeltidium free, complete keel along anterior margin, 6 tubercles posterior to keel, connected to it by single branch each. Scute 1.8 long, 1.9 wide. All keels relatively low. Small keel cells present on areas 1–4; area 1 with 2–3 small cells in midline, area 2 with 4–6 small cells in transverse row less than ½ width of scute, area 3 with 5–6 small cells in midline, area 4 with 10–12 small cells in transverse row about ½ width of scute. Paired median scute spines not prominent, on areas 4, 5 only slightly larger than adjacent keel tubercles (Fig. 46).

Chelicerae (Fig. 16) with basal article 0.70 long, 0.23 wide, sparsely setose; second article 0.72 long, 0.20 wide. Palpus (Fig. 25) slender, with dense vestiture of clavate setae; trochanter with two prominent seta-bearing ventral tubercles; dimensions given in Table 8. Legs in order of length, 2 (14.05), 4 (9.43), 3 (5.90), 1 (5.73); metatarsus 2 with about 12 false articulations, femora without false articulations; tarsi 1–4 with 6, 12, 6(7), 9 articles respectively. Lengths of leg segments given in Table 8. Length/width ratios of femora, in order: 8.25, 36.8, 9.0, 28.2. Leg femora with typical ornamentation.

Genital operculum broadly rounded, separated from sternite by suture. Ovipositor typical of genus.

**Table 8.** Appendage article measurements of Trilasma petersprousei sp. n. female.

	Femur	Patella	Tibia	Metatarsus	Tarsus
Palpus	1.00	0.86	0.70	---	0.44
Leg 1	1.65	0.60	1.08	1.15	1.25
Leg 2	4.05	0.90	3.10	3.50	2.50
Leg 3	1.80	0.50	1.30	1.05	1.25
Leg 4	3.10	1.10	2.40	1.25	1.58

###### Notes.

While Trilasma petersprousei sp. n. has long, thin legs and palpi, it does not appear in any other way to be a troglobiont; the eyes are large and well-pigmented and it lacks the exaggerated median hood process of Trilasma sbordonii, a true troglobiont.

Hoya de las Guaguas (sometimes spelled Huahuas in Spanish) is an immense pit, 478 m (1565’) deep, developed in limestone of the Sierra Huasteca, and located at 21°31'56'N; 99°02'01.15W, about 460 m (1500’) asl. The entrance is large enough that sufficient light reaches the floor to sustain a plant community (P. Sprouse, pers. comm.).

##### Trilasma chipinquensis sp. n.

urn:lsid:zoobank.org:act:6C2FDB74-23B9-4266-92F9-FE96DD129CB6

Figs 17 26 50 

###### Types.

Female holotype (AMNH) from Chipinque Mesa, Monterrey, Nuevo Léon, México, collected 24 June 1969 by Stewart B. Peck.

###### Diagnosis.

The paired median area tubercles are strongly developed in this species and project well above the level of the keels. Like trispinosum sp. n., there is a single false articulation in female femur 4, but trispinosum sp. n. has 3 lateral hood processes, while chipinquensis sp. n. has 2.

**Figures 50, 51.**
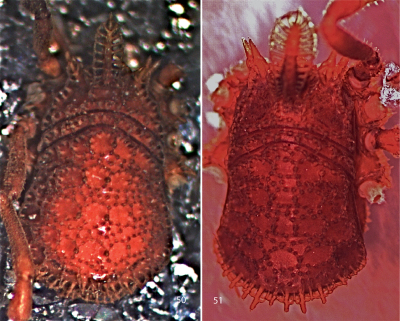
Photographs of bodies, dorsal views. **50** female Trilasma chipinquensis sp. n. **51** male Trilasma hidalgo sp. n.

###### Etymology.

The species epithet, an adjective, refers to the type locality.

###### Description.

Female holotype: total length, 2.7, width, 1.5. Color dark brown, nearly black (possibly artifact of preservation). Carapace arcuate, about 1.5X as wide as long, with complete lateral and posterior submarginal keels; pair of median keels connecting eye tubercle and innermost lateral hood process, lateral keels arising both on innermost and middle lateral hood processes. Two blunt lateral hood processes each about one-half as long as median hood process. Circumocular keels strongly developed, but subocular portion easily seen in dorsal view, eyes relatively small. Median hood process arising dorsally on eye tubercle, with nearly parallel sides, only slightly converging distally, length 0.95, width 0.40; median keels of carapace continuing as rows of lateral tubercles on median hood process, about 16 lateral tubercles, linearly connected; about 6 dorsal tubercles present, connected in a single row to one another but not to lateral tubercles. Metapeltidium free, complete keel along anterior margin, 6 tubercles posterior to keel, connected to it by single branch each. Scute 1.7 long, 1.5 wide. All keels well elevated above dorsum. Small keel cells present on areas 2–4; areas 2, 3 with 5–8 small cells in midline, area 4 with 10–12 small cells in transverse row about 2/3 width of scute. Paired median scute spines prominent, on all areas significantly larger than adjacent keel tubercles, standing well above keels; pair of spines also present on metapeltidium (Fig. 50).

Chelicerae (Fig. 17) with basal article 0.64 long, 0.21 wide, sparsely setose; distal article 0.60 long, 0.19 wide. Palpus (Fig. 26) relatively slender, with dense vestiture of clavate setae; trochanter with two prominent seta-bearing ventral tubercles; dimensions given in Table 9. Legs in order of length, 2? (-), 4 (5.62), 3 (4.66), 1 (4.20); metatarsi without false articulations, femora 4 without single false articulation; tarsi 1–4 with 5, -, 7, 6 articles respectively (tarsi 2 missing). Lengths of leg segments given in Table 9. Length/width ratios of femora, in order: 5.56, 12.86, 5.56, 7.60. Leg femora with typical ornamentation.

Genital operculum broadly rounded, separated from sternite by suture. Ovipositor typical of genus.

**Table 9.** Appendage article measurements of Trilasma chipinquensis sp. n. female.

	Femur	Patella	Tibia	Metatarsus	Tarsus
Palpus	0.80	0.32	0.30	---	0.40
Leg 1	1.00	0.40	0.64	1.20	0.96
Leg 2	1.80	0.60	1.10	---	---
Leg 3	1.00	0.46	1.00	1.20	1.00
Leg 4	1.52	0.50	1.00	1.40	1.20

###### Notes.

The holotype female was mentioned and briefly described by Shear and Gruber (1983). The present examination resulted in some different observations, primarily in that I could not see the false articulation of the second femur observed in 1983. The second leg is absent from the left side, and broken off at the tibia-metatarsus joint on the right.

Chipinque Mesa is a ridge of the Sierra Madre Oriental overlooking the city of Monterrey, to the north. Approximate coordinates are 25°36'29.43N; 100°21'18W; elevation at the top of the ridge is 1524 m (5000’). Chipinque Mesa is now a part of the Parc Nacional Cumbre and is a popular sight-seeing destination for visitors to Monterrey. It is densely forested in pines.

##### Trilasma bolivari

 Goodnight & Goodnight, 1942

Figs 52, 53

Trilasma bolivari Goodnight and Goodnight 1942, p. 7; Roewer 1940, p. 56; Shear & Gruber, 1983, p. 42.

###### Notes.

Shear and Gruber (1983) provided a detailed description based on specimens from Llano Grande, Puebla, México, a location about 28 km due northwest of the type locality, Río Frío. Now, judging from Google Earth aerial photographs, both of these places have become heavily urbanized and it seems unlikely conditions exist any longer that could support this species. Shear and Gruber (1983) mapped some of the localities given by Goodnight and Goodnight (1942, 1945b), but were not able to find all of them. Shear and Gruber expressed doubt about the identity of specimens from the Nevada de Colima, Jalisco, locality in particular; it is far separated from the others, as is the Guanajuato locality (Goodnight and Goodnight 1942). The presence of another species (see below) in Hidalgo, not far from the localities in Puebla and El Distrito Federal, suggests that at least the Guanajuato and Jalisco material may also represent undiagnosed species.

I no longer include Ruaxphilos petrunkevitchou Goodnight & Goodnight in the synonymy of Trilasma bolivari, as Shear and Gruber (1983) did, because of the distance between the type localities (that of petrunkevitchou is in Veracruz). It is very likely that petrukevitchou is another species, but the type is an early instar that has not developed any of the species-diagnostic characters. Its identity can only be established by the collection of adults at the type locality.

**Figures 52, 53.**
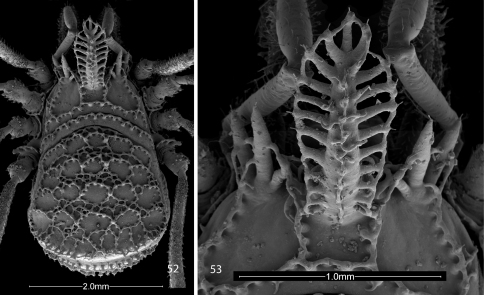
Scanning electron micrographs of female Trilasma bolivari Goodnight & Goodnight 1942 **52** body, dorsal view **53** hood, dorsal view.

I provide some new illustrations (Figs 52, 53) for comparison with the other species described here as new. The illustrations are of a female specimen collected by Fred Coyle at the pass between Toluca and México City, Distrito Federal, on Rt. 15, elev. 3000 m (9800’) asl, on 4 June 1982.

##### Trilasma hidalgo sp. n.

urn:lsid:zoobank.org:act:1CA2B4A7-FC11-471B-A1DF-BF4236BDB5B2

Figs 18 27 40, 41 51

Trilasma bolivari Goodnight & Goodnight, 1945b (not 1942), p. 8, in part only.

###### Type.

Male holotype (TMM) from El Chico, Pachuco, Hidalgo, México, collected 1 January 1976, no collector named.

###### Diagnosis.

This species is closest to Trilasma bolivari sp. n., but differs from it in having fewer small cells on the scute, shorter, stouter legs, and less prominent scute area tubercles. In addition the dorsal tubercles on the median hood process of Trilasma bolivari sp. n. are much more numerous and are scattered over the stem of the process, with connections to the lateral tubercles; Trilasma hidalgo sp. n. has fewer dorsal tubercles which are arrayed in a line and only rarely connected to the lateral tubercles.

###### Etymology.

The species epithet is a noun in apposition, referring to the Mexican state of Hidalgo.

###### Description.

Male holotype: total length, 3.2, width, 1.3. Color dark brown, nearly black. Carapace arcuate, about 1.5X as wide as long, with complete lateral and posterior submarginal keels; pair of median keels connecting eye tubercle and innermost lateral hood process, lateral keels arising both on innermost and middle lateral hood processes. Two blunt lateral hood processes each about one-third as long as median hood process. Circumocular keels strongly developed, subocular portion especially prominent. Median hood process arising dorsally on eye tubercle, widest point past midline of length, length 1.0, width 0.4; median keels of carapace continuing as rows of lateral tubercles on median hood process, about 30 lateral tubercles, linearly connected; about 15 dorsal tubercles present, connected in a single row to one another but not to lateral tubercles. Metapeltidium free, complete keel along anterior margin, 8 tubercles posterior to keel, connected to it by single branch each. Scute 1.3 long, 1.3 wide. All keels well elevated above dorsum. Small keel cells present on areas 2–5; area 2 with 5 or 6 small cells in single transverse row; area 3 with 10 to 12 small cells in single transverse row, but row is slightly wider than row on area 2; area 4 with two paramedian groups of 2 or 3 small cells each; area 5 similar, but only 1 or 2 small cells. Paired median scute spines prominent, significantly larger than adjacent keel tubercles on areas 2–5 (Fig. 51).

Chelicerae (Fig. 18) with basal article 0.82 long, 0.28 wide, sparsely setose; second article 0.65 long, 0.22 wide. Palpus (Fig. 27) stout, tibia, patella crassate, with dense vestiture of clavate setae; trochanter with two prominent seta-bearing ventral tubercles; dimensions given in Table 10. Legs in order of length, 2 (7.44), 4 (5.70), 3 (4.01), 1 (3.98); no false articulations; tarsi 1–4 with 4, 5, 5, 6 articles respectively. Lengths of leg segments given in Table 10. Length/width ratios of femora, in order: 4.58, 12.50. 4.58, 8.0. Leg femora with typical ornamentation.

Genital operculum broadly rounded, not separated from sternite by suture. Penis typical of genus (Figs 40, 41).

**Table 10.** Appendage article measurements of Trilasma hidalgo sp. n. male.

	Femur	Patella	Tibia	Metatarsus	Tarsus
Palpus	0.80	0.56	0.44	---	0.37
Leg 1	1.10	0.60	0.80	0.70	0.78
Leg 2	2.00	0.70	1.70	1.60	1.44
Leg 3	1.10	0.56	0.90	0.65	0.80
Leg 4	1.60	0.60	1.60	0.90	1.00

###### Notes.

“El Chico” doubtless refers to what is now Parque Nacional El Chico, located north of the city of Pachuca. The Parque is extensive but approximate coordinates are 20°12'26N; 98°43'52W; elevations within the park range from 2300–3090 m (7540–10131’) asl. The mountains are covered with a dense pine forest, with fir at the higher elevations. Goodnight and Goodnight (1945b) reported Trilasma bolivari from three separate collections at this place; those specimens (AMNH, not re-examined for this study) are undoubtedly hidalgo.

##### Trilasma tropicum sp. n.

urn:lsid:zoobank.org:act:58A227AB-0922-4C73-A739-510A54F7732F

Figs 19 28, 29 38, 39 49 54, 55 

###### Types.

Male holotype and female paratype from “Las Ventas, Honduras,” collected 11 February 1939 by R. V. Chamberlin (AMNH).

###### Diagnosis.

The short, relatively crassate legs and the extremely prominent subocular keels (Fig. 55) separate this species from others. Trilasma ranchonuevo sp. n. (Tamaulipas, México) has false articulations in femora 2 and 4 that are absent in Trilasma tropicum sp. n. The male has an acute dorsal knob on the basal article of the chelicerae, as well as the tooth on the second article.

**Figures 54, 55.**
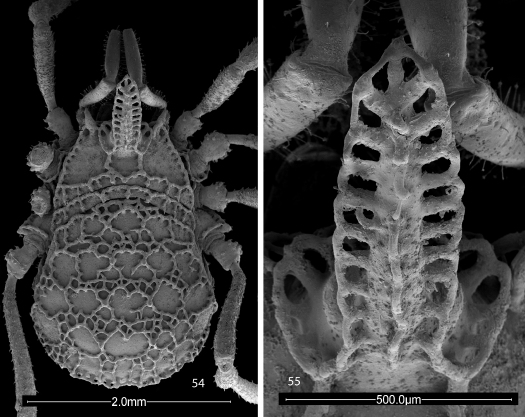
Scanning electron micrographs of female Trilasma tropicum sp. n. **54** body, dorsal view **55** eye tubercle, dorsal view.

###### Etymology.

The species epithet refers to the occurrence of the species in the Neotropics.

###### Description.

Male holotype: total length, 2.5, width, 1.4. Color uniform light chestnut brown, metatarsi and tarsi of legs 3, 4 whitish yellow. Nonsexual characters as in female (see Figs 54, 55, and description, below), but dorsal ornament reduced, partially obscured in holotype by secretion; median hood process 0.80 long, 0.3 wide. Scute 1.4 long, 1.4 wide.

Chelicera (Fig. 19) without gland on basal article but with dorsal, conical protuberance, second article with forward-projecting tooth, basal article 0.50 long, 0.21 wide; distal article 0.55 long, 0.19 wide. Palpal patellae and tibiae swollen, epigamic glands probably present but not marked by patches of fine setae (Fig. 29), dimensions of palpus given in Table 11. Legs in order of length, 2 (6.44), 4 (5.34), 3 (3.76) , 1 (3.80); legs lacking false articulations; tarsi 1–4 with 5, 5, 6, 7 articles respectively. Lengths of leg segments given in Table 11. Length/width ratios of femora, in order: 4.5, 10.7, 4.2, 7.8.

Genital operculum broadly rounded, marginate, with two small, lateral notches. Penis typical (Figs 38, 39).

Female paratype: The female paratype (Figs 54, 55) is mounted on a SEM stub, therefore it was not possible to get accurate measurements of the appendages. Total length, 2.9, width, 1.8. Carapace arcuate, about twice as wide as long, with complete lateral and posterior submarginal keels; pair of median keels connect eye tubercle and innermost lateral hood process, lateral keels also arising on innermost lateral hood process. Two blunt lateral hood processes each about one-fourth as long as median hood process. Circumocular keels prominent, subocular portion strongly developed, eyes of usual size. Median hood process arising rostrally on eye tubercle, relatively narrow, length 0.8, width 0.3, ten fenestrations on each side, sides nearly parallel; median keels of carapace continue as rows of reduced lateral tubercles on median hood process, about 20–22 lateral tubercles, linearly connected; about 8 dorsal tubercles present, connected to each other but not to lateral tubercles. Metapeltidium free, complete keel along anterior margin, 10 tubercles posterior to keel, connected to it by single branch each. Scute 1.7 long, 1.7 wide. All keels relatively low. Small keel cells of scute area 1 present, small keel cell rows progressively wider posteriorly, widest on area 4; small cells of area 5 few, in two small groups near midline. Paired median scute spines moderately developed, on areas 4 distinctly larger than adjacent keel tubercles.

Chelicerae as described for male, but lacking dorsal protuberance on basal article, tooth on second article. Palpus (Fig. 28) as in male but patellae and tibiae not swollen. Legs in order of length, 2, 4, 3, 1; leg articles without false articulations. Leg femora with typical ornamentation.

Genital operculum broadly rounded, marginate, with suture.

**Table 11.** Appendage article measurements of Trilasma tropicum sp. n. male.

	Femur	Patella	Tibia	Metatarsus	Tarsus
Palpus	0.72	0.60	0.46	---	0.30
Leg 1	0.90	0.46	0.64	0.90	0.90
Leg 2	1.50	0.56	1.08	2.00	1.30
Leg 3	0.84	0.50	0.66	0.80	0.96
Leg 4	1.40	0.56	1.28	1.00	1.10

###### Notes.

I mounted the only female on an SEM stub under the impression there was a second female in the collection, but the second specimen turned out to be a male. Thus measurements of the appendages of the female became impossible, but there is no reason not to suspect that the typical sexual dimorphism in appendage lengths is characteristic of this species.

Las Ventas is not listed for Honduras by the U. S. Board on Geographical Names (http://geonames.nga.mil/ggmagaz), nor could it be found by Google Earth. There are four places named Las Ventanas and one called Las Ventanillas, scattered in four different departments of Honduras. There is a village of Las Ventas in El Salvador (13°35'58.55N; 88°21'00W; 365 m (1050’) asl) but it is in the central part of the country, not close to the border with Honduras. Otherwise the name evidently is not used for a populated place in Central America. The type locality is therefore in doubt, but because the collection was made 70 years ago, it is possible “Las Ventas” was in existence then in Honduras, having since disappeared from geographic databases, or the label is a lapsus for one of the Las Ventanas. In any case, if this species is really from Honduras, it occurs farther south than any New World dyspnoan, at approximately the same latitude as Cladolasma angka in Thailand.

## Supplementary Material

XML Treatment for Ortholasmatinae
